# Allosteric Regulation in the Ligand Binding Domain of Retinoic Acid Receptorγ

**DOI:** 10.1371/journal.pone.0171043

**Published:** 2017-01-26

**Authors:** Yassmine Chebaro, Serena Sirigu, Ismail Amal, Régis Lutzing, Roland H. Stote, Cécile Rochette-Egly, Natacha Rochel, Annick Dejaegere

**Affiliations:** 1 Department of Integrative Structural Biology, Institut de Génétique et de Biologie Moléculaire et Cellulaire (IGBMC), Institut National de la Santé et de la Recherche Médicale (INSERM) U964, Centre National de la Recherche Scientifique (CNRS) UMR 7104, Université de Strasbourg, Illkirch, France; 2 Department of Functional Genomics and Cancer, Institut de Génétique et de Biologie Moléculaire et Cellulaire (IGBMC), Institut National de la Santé et de la Recherche Médicale (INSERM) U964, Centre National de la Recherche Scientifique (CNRS) UMR 7104, Université de Strasbourg, Illkirch, France; Russian Academy of Medical Sciences, RUSSIAN FEDERATION

## Abstract

Retinoic acid (RA) plays key roles in cell differentiation and growth arrest through nuclear retinoic acid receptors (RARs), which are ligand-dependent transcription factors. While the main trigger of RAR activation is the binding of RA, phosphorylation of the receptors has also emerged as an important regulatory signal. Phosphorylation of the RARγ N-terminal domain (NTD) is known to play a functional role in neuronal differentiation. In this work, we investigated the phosphorylation of RARγ ligand binding domain (LBD), and present evidence that the phosphorylation status of the LBD affects the phosphorylation of the NTD region. We solved the X-ray structure of a phospho-mimetic mutant of the LBD (RARγ S371E), which we used in molecular dynamics simulations to characterize the consequences of the S371E mutation on the RARγ structural dynamics. Combined with simulations of the wild-type LBD, we show that the conformational equilibria of LBD salt bridges (notably R387-D340) are affected by the S371E mutation, which likely affects the recruitment of the kinase complex that phosphorylates the NTD. The molecular dynamics simulations also showed that a conservative mutation in this salt bridge (R387K) affects the dynamics of the LBD without inducing large conformational changes. Finally, cellular assays showed that the phosphorylation of the NTD of RARγ is differentially regulated by retinoic acid in RARγWT and in the S371N, S371E and R387K mutants. This multidisciplinary work highlights an allosteric coupling between phosphorylations of the LBD and the NTD of RARγ and supports the importance of structural dynamics involving electrostatic interactions in the regulation of RARs activity.

## Introduction

Retinoic Acid Receptors (RARs) belong to the large family of nuclear receptor proteins (NRs). RARs are ligand-dependent transcription factors involved in a plethora of cellular phenomena from embryonic development and organogenesis to homeostasis of most adult tissues [1–5]. Their activity is controlled by retinoic acid (RA), the major active metabolite of Vitamin A (retinol). There are three RAR subtypes, RAR α, β and γ [6], with several isoforms (α1, α2, γ1, γ2, etc.) in vertebrates. Forming heterodimers with Retinoid X Receptors (RXRs) [7, 8], RARs regulate the expression of several target genes via binding specific retinoic acid response elements (RARE) [3, 9].

RARs display a common NR modular organization with mainly two structured domains, a central DNA binding domain (DBD), a C-terminal ligand binding domain (LBD), and an unstructured N-terminal domain (NTD). Crystallographic and Nuclear Magnetic Resonance (NMR) analysis of the DBD and of the LBD, coupled to the characterization of the associated multi-protein complexes, provided insights into the molecular mechanism of transcription regulation by RARs (reviewed in [10]). Indeed, upon ligand binding, conformational changes occur in the LBD, with subsequent modulation of the interaction network of the receptors [11–13] Computational tools have also proven to be useful in understanding the allosteric regulation [14] that takes place in NRs [15], for instance upon ligand binding and co-activator binding site formation [16] as well as between the ligand and a co-activator peptide [17].

Despite high sequence homology (97–100% identity in the DBD and 85–90% in the LBD [18]), shared structural organization, and common physiological ligands, the different RAR subtypes display specific and distinct regulatory roles [19–22]. In view of the physiological importance of RARs, deciphering details of their specific regulatory pathways remains of considerable fundamental and pharmacological importance [22].

**Fig 1:**
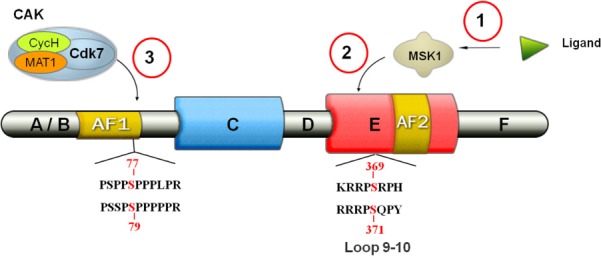
Modular organization of RARs and representation of the phosphorylation sites within the NTD and LBD. First, through non-genomic effects, RA activates MSK1, which then phosphorylates the LBD (S369 in RARα and S371 in RARγ). Then, the CAK complex is recruited to the LBD via cyclin H, allowing the phosphorylation of the NTD by cdk7 (S77 in RARα and S79 in RARγ).

Like other NRs, RARs are also phosphoproteins [23] and recent evidence indicates that, in addition to classical transcriptional effects, RA also induces extra-nuclear and non transcriptional responses such as the rapid and transient activation of kinase cascades [24]. These RA-activated kinases then translocate into the nucleus where they phosphorylate several targets including RARs [24]. The molecular details of the cross-talk between the non-genomic and genomic effects of RARs are still far from being understood. However, several studies have shown that RARs contain phosphorylation sites [25] in the LBD (S369 in RARα1 and S371 in hRARγ1) and in the NTD (S77 in RARα1 and S79 in RARγ1). Moreover, phylogenetic analysis showed that these sites are conserved in the different mammalian RAR subtypes [26] (Fig 1).

Recent studies showed that, in response to RA, RARα is rapidly phosphorylated at S369 located in loop L9-10 within the LBD (Fig 1) [27]. This phosphorylation then induces activation of an allosteric communication pathway that affects the nearby loop, L8-9, which corresponds to the cyclin H docking site [26, 28]. Consequently, phosphorylated RARα can better bind the cyclin H/cdk7/CAK sub-complex of the general transcription factor TFIIH [27], leading to the phosphorylation by cdk7 of S77 located in the NTD of the receptor (Fig 1) [29, 30]. This last phosphorylation is crucial for the control of RARα target genes transcription [27].

Interestingly, the phosphorylation of the RARγ NTD also involves cdk7 [30] and is also mandatory for the transcription of the RARγ-target genes [31]. In the present study, we aimed at determining whether RARγ phosphorylation is regulated by an allosteric pathway similar to that observed for the RARα subtype. To do so, we first solved the crystallographic structure of a RARγ LDB mutant mimicking the receptor phosphorylated at position S371 (S371E). We then analyzed its structure and dynamics by molecular dynamics simulations. We found that the S371E mutation affects distant electrostatic interactions in the LBD, in particular, in the vicinity of the cyclin H binding site. We identified a salt bridge (involving R387) whose dynamics differ in the wild-type and S371E conformational ensemble. The mutant R387K was prepared and tested in cellular assays, which showed that the phosphorylation of the RARγNTD regulated by retinoic acid is differentially modulated in RARγWT versus the mutants. The results show that destabilization of the R387 salt bridge abolishes phosphorylation of the NTD, supporting its role as a crucial bridge in the allosteric communication pathway.

## Materials and Methods

### Purification and crystallization of the human RARγ LBD complexed with 9cis RA

These experiments were done as previously described [32]. The LBD of the human RARγ Ser371Glu mutant (residues 178–423) was subcloned in the pET15b expression vector to obtain an N-terminal hexahistidine-tagged fusion protein and was overproduced in *E*. *Coli* BL21 (DE3). Cells were grown in LB medium containing 5% sucrose and subsequently induced for 4 h at 25°C with 1mM isopropyl thio-β-D-galactoside. Purification included a metal affinity chromatography step on a nickel-chelating resin. After tag removal by thrombin digestion, the protein was further purified by gel filtration on HiLoad 16/60 Superdex 75 (GE Healthcare). The final buffer contained 10mM Tris, pH7.5, 100mM NaCl, and 5mM dithiothreitol. Then 9-cis RA was added in a 2-fold excess to the purified receptor in order to ensure full saturation. Purity and homogeneity of the protein were assessed by SDS-PAGE and Native-PAGE. In the case of the S371E mutant, crystals were obtained at 17°C by the hanging drop vapour diffusion technique. The reservoir buffer contained 0.2 M Calcium Chloride, 0.1 M, Tris HCl pH 7.5, 14% Peg 6K.

### X-ray data collection and refinement

The crystals were mounted in fiber loops and flash cooled in liquid ethane at liquid nitrogen temperature after cryoprotection. Data collection from a single frozen crystal was performed at 100K at beamline ID14-1 at 100 K at ESRF (Grenoble, France). The crystals were isomorphous and belonged to the space group P4_1_2_1_2 with the unit cells parameters as specified in S1 Table. Data were integrated and scaled by using the HKL2000 program package[33]. The crystal structure of the RARγ S371E complex was solved by molecular replacement using the hRARγ wild type LBD structure (pdb id 3LBD) [34] as a starting model using AMoRe[35] integrated in the graphical interfaced version of CCP4 (Collaborative Computational Project, 1994). Manual building of the structure was carried out with COOT[36] with iterative cycles of structure refinement performed through REFMAC5[37, 38].The parameters and the topology coordinate for the ligand and the detergent were generated from PRODRG[39]. The final steps of the refinement included the addition of the ligand, the detergent and the water molecules. The refined model showed unambiguous chirality for the ligand and no Ramachandran plot outliers according to PROCHECK[40]. For the structure comparison, Cα traces of the models were superimposed using the LSQ commands of O and default parameters. The structure is deposited in the Protein Data Bank under the accession code 5M24.

### Molecular dynamics simulations

Simulations were performed with the LBD of RARγ WT (pdb id 3LBD)[34] and S371E (using the crystal structure obtained in this study). Substitution of the R387 residue by a lysine in the wild-type structure was performed using the mutagenesis wizard in the PyMol program[41]. In total, three sets of starting structures for molecular dynamics simulations were prepared. Histidine protonation states were calculated at physiological pH 7.4 based on the protein structure using PROPKA[42, 43]. Hydrogen atom placement was performed using the HBUILD[44] facility in the CHARMM program[45]. The wild type and mutant LBDs were solvated in cubic boxes of approximately 76Å per side, with a salt concentration Na+/Cl- corresponding to the physiological concentration of 0.15M. Prior to solvating the systems, two sequential minimizations of 100 steps Steepest Descent method and 1000 steps of Adapted Basis Newton-Raphson method were performed in order to eliminate strong steric contacts.

Molecular dynamics simulations were realized using the CHARMM27 force field[46] within the NAMD program[47]. Each system was prepared for simulation in two steps: 1. minimization and heating of water molecules around the fixed protein; 2. minimization and heating of the entire system. The first step consisted of 1000 steps of Conjugated Gradient (CG) energy minimization, heating up to 600K over 23ps, 250 steps of CG energy minimization, and again heating to 300K over 25ps. In the second step, positional restraints on the protein were removed and the entire system was energy minimized by 2000 steps of CG and then heated to 300K over 15ps, followed by 85ps of equilibration. The production run was then performed for 100ns. Periodic boundary conditions were used and the particle mesh Ewald algorithm[48] was applied to take into account long-range electrostatic interactions. All bonds between heavy atoms and hydrogens were constrained using the SHAKE algorithm[49] and an integration time step of 1fs was used for all simulations. The whole protocol was repeated four times for each structure of RARγ, (WT, S371E and R387K mutants), resulting in a total simulation time of 400ns per structure. Time evolution of the backbone RMSD suggests no critical change in the conformations of the protein and remains stable between 1 and 2Å throughout all simulations (S1 Fig).

### Analysis of the trajectories

The first 30 ns of all molecular dynamics simulations were excluded from the analysis in order to ensure that the analysis was done over the equilibrated system. As part of our analysis of the molecular dynamics simulations, we define and calculate an electrostatic contact interaction according to the following criterion: a contact is defined if any oxygen atom of an acid residue side chain lies within 3.5Å of any nitrogen atom of a basic residue side chain. The electrostatic contact probabilities were obtained by dividing the number of contacts per residue by the number of trajectory frames analyzed. In addition to these probabilities, we calculated the distances between any oxygen and nitrogen atom in the side chain of oppositely charged residues in order to be able to calculate the statistical significance of the results. The atoms considered were namely Nε, Nη1 and Nη2 of Arg, Nζ of Lys, Oδ1, Oδ2 of Asp and Oε1, Oε2 of Glu. We then performed a Student’s t-test to assess the relevance of the calculated means. Distribution plots of these distances and the corresponding averages are provided in S2 and S3 Figs for residues around the phosphorylation site and the cyclin binding site respectively.

We further analyzed the structure and relative orientation of the helices of the LBD. Angles between two helices were calculated using the Chothia-Levitt-Richardson algorithm[50] implemented in the CHARMM program. The cylinder most approximating each helix was determined based on the Cα-atoms. The maximum angle of bending within the helices was calculated using the BENDIX plugin[51] in VMD[52], calculating bendices every 4 residues. The accuracy of the calculated means for the angles between helices and angles of bending were evaluated through bootstrapping using 1000 bootstrap replicates, and the standard errors calculated on all averages were less than 0.009 in all data sets. We performed a Student’s t-test to evaluate the statistical significance of the differences in the compared averages in the angles between helices and the angles of bending within helix H9.

### Analysis of RARγ phosphorylation

hRARγ1WT was subcloned from the pSG5 construct[30] into the pcDNA3.1 vector with added Flag and HA tags. hRARγS371E, R387K and S371N in the same pcDNA vector were constructed by double PCR amplification reactions. The constructs were introduced into mouse embryo fibroblasts (MEFs) with all three RARs deleted (RAR(α, β, γ)-/-triple RAR knockout cells)[27] using the X-tremeGENE HP DNA Transfection reagent (Roche). When 80–90% confluent, cells were treated with RA (10–7 M; Sigma Aldrich Corporation) after 24 hours in medium containing 1% dextrancharcoal- treated fetal calf serum. Whole cell extracts were prepared and immunoprecipitated with purified mouse monoclonal antibodies recognizing RARγ phosphorylated at S79 (Ab 14γ)[53] and immobilized on Dynabeads Protein G (Invitrogen). Then the eluates were analyzed by immunoblotting with rabbit polyclonal antibodies against RARγ (RPg(F))[53]. Phosphorylation blots were repeated three times and a representative one among the three was selected for the corresponding figure.

## Results and Discussion

### Crystal structure of hRARγS371E (LBD) bound to 9-cis retinoic acid

**Fig 2:**
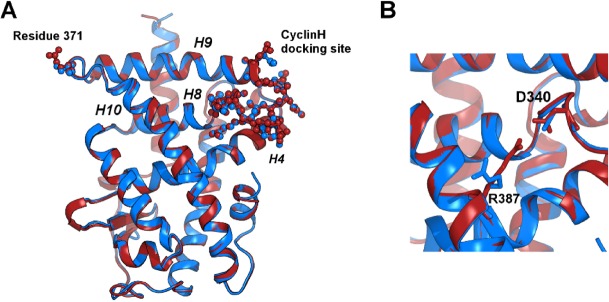
Superposition of RARγ WT (in blue, pdb code: 3LBD) and RARγS371E (in red) crystal structures. (A) Residue 371 and the cyclin H docking site are represented in spheres and sticks. (B) The main difference between the two structures is the orientation of the R387 side chain.

The hRARγ LBD crystallized in presence of the neutral detergent n-dodecyl-β-d-maltopyranoside (C12m) that occupies the coactivator binding cleft of the receptor [54]. The S371 phosphorylation site of RARγ lies in the surface-exposed loop L9-10, which is easily accessible to cellular protein kinases. To investigate the consequences of S371 phosphorylation, we crystallized a hRARγ LBD mutant bound to 9cis RA and C12m in which Ser371 is substituted by a Glu residue, which mimics a phosphorylated residue (hRARγS371E LBD). The complex crystallized into the P4_1_2_1_2 with one monomer per asymmetric unit. The complex structure was solved by molecular replacement and refined to 1.7Å. Refinement statistics are summarized in S1 Table. The hRARγS371E-holo-LBD adopts the classical anti-parallel alpha helical "sandwich" similar to that of the previously described wild type hRARγ LBD structure conformation [55]. The electron density map showed the presence of the ligand buried into the ligand-binding pocket and an extra electron density around position 371 confirmed the mutation of the serine residue into a glutamic acid. The detergent mediates a crystal contact in the region of the receptor involved in coactivator binding as described for the RARγ wild-type [54]. The hRARγS371E and hRARγWT LBD structures are isomorphous and very similar with a Cα-RMSD of 0.47Å (Fig 2A). The only notable difference between the two structures is the orientation of Arg387 in H10 (Fig 2B). Indeed, in RARγ wild-type R387 points towards the solvent (Fig 2B), while in RARγS371E it forms a salt bridge with Asp340.

### The phospho-mimetic residue modifies the electrostatic interactions and dynamics in RARγLBD

To further understand the effects on allosteric signaling of substituting S371 by a glutamic acid in the RARγ LBD, we performed molecular dynamics (MD) simulations of both the WT and S371E structures bound to 9cis RA (see Materials and Methods). Conserved salt bridges in the LBDs of nuclear receptors are well characterized and are used to define different classes of nuclear receptors [56]. Therefore, we analyzed the salt bridge networks in the conformational ensemble collected from the MD simulations and identified those interaction pairs that are altered in the S371E mutant.

**Fig 3:**
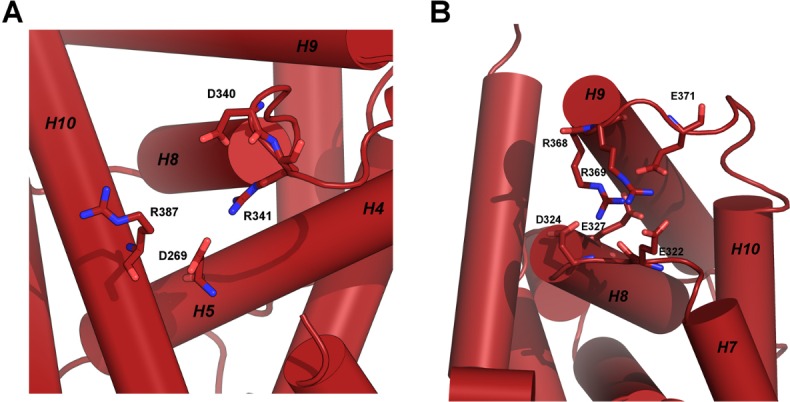
Representation of the electrostatic networks in the LBD of RARγ. The side chains of residues D269, D340, R341, R387 are represented around the cyclin H docking site (A) and side chains of residues E322, D324, E327, R368, R369 around the phosphorylation site (B).

First, we investigated whether the substitution of S371 by a glutamic acid in loop L9-10 affects the salt bridge networks around S371, which is located in a region rich in charged amino acids (Fig 1). In RARγWT, R368 and R369 (Fig 3B) interact electrostatically with E322, D324 and E327. This network of interactions is dynamic, in that the R and D/E residues implicated in ionic interactions are able to exchange interaction partner residues over the course of the simulations. To characterize the electrostatic network, we calculated the probability of forming an electrostatic contact over the course of the simulations, as defined when any oxygen atom of an acidic residue side chain lies within 3.5Å of any nitrogen atom of a basic residue side chain.

The negative charge introduced by E371 in loop L9-10 leads to ionic contacts with R369 in 11% of simulation frames analyzed from the mutant simulations compared to 0% in the WT ones. Distance distributions between N atoms of R369 side chain and O atoms of E371 side chain were compared to the distances with the Oγ atom of S371 (S2 Fig) in the mutant and WT simulations respectively. The distributions show that the distances are shifted towards smaller values in the S371E mutant with respect to the WT.

Comparison of the dynamics of the WT and S371E LBDs showed that fewer contacts in the vicinity of the mutation are made in the mutant compared to the native structure (Table 1). This suggests a loss of stability of the electrostatic network around the mutated site. In addition, we also calculated all distances between nitrogen atoms of Arg and Lys side chains and oxygen atoms of Glu and Asp acidic side chains as discussed in Materials and Methods. The histograms of the distances around the phosphorylation site (Table 1) and their corresponding averages are represented in S3A and S3B Fig. These results show that percentages of distances that are between 2 and 4Å for salt-bridges parteners are modified in the S371E mutant simulations with respect to the WT. In particular, the R368-D324 salt bridge is significantly less frequently formed in the mutant, in agreement with the electrostatic contact probabilities (see Table 1) discussed previously.

**Table 1:** Summary of structural properties calculated from the MD simulations of RARγ wild-type, S371E and R387K.

	Wild-type	S371E	R387K
Residue pair^**1**^			
R368-D324	0.6	0.14	0.34
R368-E327	0.94	0.9	0.82
R369-E322	0.33	0.24	0.07
R369-E327	0.21	0.1	0.51
TOTAL	*2*.*08*	*1*.*38*	*1*.*74*
R/K387-D340	0.52	0.84	0.25
R341-D269	0.99	0.88	0.93
Angles between helices^**2**^			
H9-H10	47.6	49	46.9
H9-H8	13.3	15	14.5
H9-H4	42.9	47.3	47.1
Average maximum angle within H9^**3**^	17.2	15.4	17.7

^1^The fraction of MD simulation frames where an electrostatic contact is calculated. An electrostatic contact is defined if the distance between an oxygen atom and a nitrogen atom of acid and basic side chains respectively is below 3.5Å.

^2^Average angle values.

^3^Average value of the maximum angle calculated along the helix H9.

Next, we investigated the effects of the S to E substitution on the region previously identified as the cyclin H binding site in RARα [27, 28, 57]. This region is located over 40 Å from residue 371 (Fig 2A), and also shows a strong network of electrostatic interactions involving two salt bridges: one between R387 and D340 and another one between R341 and D269 (Fig 3A). In the crystal structures, the salt bridge between R387 and D340 is formed in RARγS371E but not in the WT LBD [54]. However, the molecular dynamics simulations suggest that there is a dynamic equilibrium of this salt bridge. Indeed, in the simulations of the WT LBD, the R387-D340 salt bridge was formed in 52% of the frames (see Table 1). That this salt bridge is dynamic in the WT LBD was further confirmed by a systematic analysis of all PDB structures of the RARγ LBD. In total, nine structures of the receptor are deposited and in six of the RARγWT structures in complex with agonists, R387 forms the salt bridge observed in the present RARγS371E structure. However, according to the electron density of these structures, D340 is more flexible and the salt bridge therefore appears weaker than in RARγS371E. The simulations of the S371E LBD showed that the equilibrium is displaced towards formation of the electrostatic interaction. Indeed, the R387-D340 salt bridge is formed in 84% of the mutant structures extracted from the simulations (Table 1). Similarly to the analysis of electrostatic contacts in the vicinity of the phosphorylation site, we calculated all distances between O and N atoms of oppositely charged residue side chains in the cyclin H docking site and the corresponding averages (S4A and S4B Fig). Histograms represented in S4 Fig are coherent with the contact probabilities and show an increase in small values of distance for R387-D340 in the S371E conformational ensemble. This shows a reinforcement of this interaction upon introduction of a negative charge at position 371, about 40Å away in the LBD. On the other hand, the second salt bridge situated in the vicinity of the cyclin binding site (R341-D269) does not show significant differences between the mutant and WT (Table 1 and S4 Fig), in agreement with the crystallographic data.

**Fig 4:**
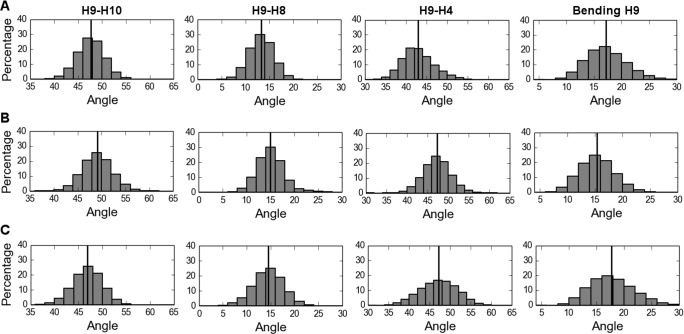
Distributions of angles between H9-H10, H9-H8, H9-H4 and bending within H9. The angle values are calculated in the simulations of the WT (A), the S371E mutant (B) and the R387K mutant (C) and their respective means are represented in horizontal black lines.

Finally, we found that the alterations in the interaction network of helix H9, both around its C-terminal part (residue 371) and around its N-terminal part (cyclin H docking site), affect the relative motions of the different helices of the receptor. We focused in particular on the angles between helices H9 and H10 (around residue 371), and between helices H9, H8 and H4 (around the cyclin H docking site) (Fig 2A). The distribution of the angle values and the corresponding average values are represented in Fig 4 and summarized in Table 1. The shift of the distributions shows that these angles are systematically larger in the S371E LBD than in the WT LBD (Fig 4A and 4B). Bending within the H9 helix was also analyzed and the angular distributions indicated that the S371E mutation results in a straighter H9 (Fig 4A and 4B).

All the distances, angles and bending averages were assessed by a Student’s t-test; the calculated p-values in the data sets were lower than 2.2e^-16^, supporting statistical significance of the differences. Together, these results indicate that the presence of a glutamic acid at position 371 in the LBD of RARγ changes the electrostatic network around L9-10 by decreasing the number of interactions with respect to the WT conformational ensemble. Consequently, the angle between helices H9 and H10 is increased and the dynamics throughout the helices are modified. Indeed, the salt bridge between D340 in loop L8-9, which underlies the cyclin H binding site, and R387 in H10 is reinforced with a concomitant increase in the angles between H9 and H8 and H9 and H4. This is, in turn, associated with the straightening of helix H9. It must be noted that these changes in conformational equilibria are subtle, and that, in agreement with the crystallographic data, no large conformational change is observed during the simulations, as shown by the rather small fluctuations in RMSD around the starting structures of each simulation (S1 Fig).

### Mutation of R387 confirms the importance of salt bridge dynamics

To investigate the role of the R387-D340 salt bridge dynamics in the allosteric communication, we constructed a mutant in which R387 is substituted by a lysine residue (see Materials and Methods). This substitution (R387K) has a minimal physico-chemical impact on the receptor, but is expected to selectively displace the equilibrium of the salt bridge towards the open form. As the arginine-to-carboxylate interactions are stronger than the lysine-carboxylate interactions [58, 59], we expected that the R/K mutation would affect the dynamic equilibrium of L8-9 corresponding to the cyclin H binding site. The conformational dynamics from MD simulations of the R387K LBD were analyzed and compared to that of the WT and S371E LBDs. As anticipated, in the R387K mutant, the salt bridge with D340 is destabilized, as measured by the percentage of electrostatic contacts (Table 1). Note that, in this mutant, the salt bridge was still present but significantly less often (25% of the simulation time) compared to the WT LBD (52%) and the S371E LBD (84%). We also calculated all distances between O and N atoms of D and R side chains respectively. Histograms of these distances are presented in S4 Fig and corroborate the decrease in contact probability in the R387K mutant.

We then investigated whether changes in dynamics of the K387-D340 salt bridge are transmitted to other parts of the LBD. We observed that, in the vicinity of the phosphorylation site at position 371, which is 40 Å from the R/K mutation site, the electrostatic contacts are altered. In particular, the salt bridge between R368 and D324, is significantly less present than in the WT (Table 1 and S3C Fig). Interestingly, this salt-bridge was also the most affected by the S371E mutation (Table 1 and S3B Fig). The formation of the other salt bridges in this region (Table 1 and S3C Fig) are also affected, and collectively the network is weaker than in the WT (Table 1), but stronger than in the S371E mutant, where the perturbation is nearby. Changes also occur in the angles between helices, where there is a shift in the distributions of the angles between helices H9-H8 and H9-H4 (Table 1 and Fig 4) towards larger values and a decrease in the angles between helices H9 and H10.

As previously discussed in the case of the WT and S371E simulations, the Student’s t-test resulted in p-values less than 2.2e^-16^ when calculated on all averages cited above, so they can be considered statistically different. Altogether these results support the idea that the R/K mutation affects the equilibrium dynamics without inducing major conformational changes.

### Crucial role of S371 phosphorylation and of the R387-D430 salt bridge in the phosphorylation of the NTD

**Fig 5:**
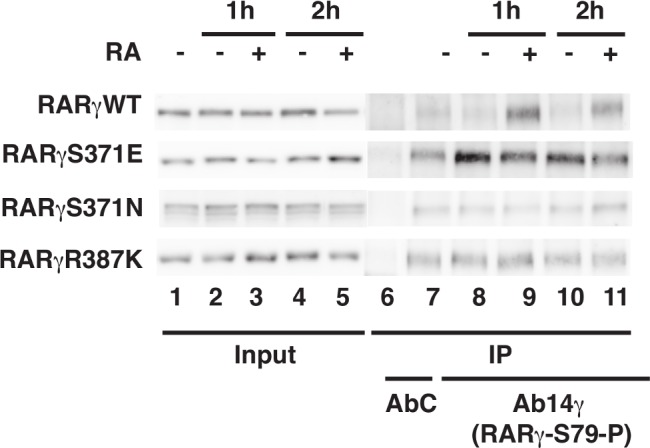
The phosphorylation of the RARγ NTD relies on the integrity of the LBD. Kinetic experiments show that RA induces the phosphorylation of RARγWT at S79 in MEFs. Phosphorylation of S79 becomes RA independent when S371 is substituted by a glutamic acid (E) residue, which is a phospho-mimetic. In contrast, S79 does not become phosphorylated when S371 is substituted by an asparagine (N) and when R387 is substituted by a lysine (K). Cell extracts were immunoprecipitated with antibodies recognizing specifically RARγ phosphorylated at S79 (Ab14γ). The eluates were resolved by SDS-PAGE and immunoblotted with RPγ(F).

Using an approach employing immunoprecipitation experiments performed with antibodies that specifically recognize RARγ phosphorylated at residue S79 in the NTD [53], we first demonstrated the existence of a phosphorylation cascade in the RARγ paralog. We found that, upon addition of RA, the phosphorylation of S79 in the RARγWT increased rapidly within 1 hour (see Fig 5, upper panel, compare lanes 8 and 9 and lanes 10 and 11). Similar observations were made for the RARα subtype [27]. This work further showed that, in RARγS371E, S79 is constitutively phosphorylated both in the absence or presence of RA (Fig 5, second layer of panels). In contrast, when S371 is substituted by an asparagine, which cannot be phosphorylated (RARγS371N), the phosphorylation of S79 does not increase in response to RA (Fig 5, third panel from the top). Remarkably, a similar observation is made for the R387K mutant, where the phosphorylation of S79 does not increase with addition of RA (Fig 5 lower panel). These results corroborate that phosphorylation of S371 is required for the phosphorylation cascade and further indicates that the dynamics of the R387-D340 salt bridge is critically involved in the signaling cascade.

## Conclusions

In the present study, we used a multidisciplinary approach combining x-ray crystal structure determination, molecular dynamics simulations and *in vitro* phosphorylation experiments to investigate the allosteric regulation that occurs upon a phospho-mimetic mutation in the LBD of RARγ. The crystal structure of the S371E variant of RARγ was solved to 1.7Å and showed that the introduction of the point mutation did not lead to any significant conformational changes. The crystal structure of the S371E mutant was very similar to that of the WT receptor [34]; the Cα-RMSD with respect to the wild-type protein was only 0.47Å and the only notable difference was the formation of a salt bridge between R387 and D340 in the mutant. This structure was used in molecular dynamics simulations, which revealed altered dynamics of the S371E LBD with respect to the WT. More specifically, the simulations of the RARγ LBD showed that the introduction of the phospho-mimetic Glu modulates ionic pair interactions that modify the relative orientations between helices and induce subtle changes in the conformational ensemble explored by the LBD. The presence of a local negative charge in the phospho-mimetic E371 led to an increase of ionic contact with R369 in its vicinity and modulates the nearby electrostatic network. The consequences of introducing this negative charge around loop L9-10 can be summarized as follows: i) a decrease in the electrostatic interactions in this region, ii) an increase in the angles between helices H9 and H10, iii) propagation of these modifications to the cyclin H binding site located 40 Å from the modified serine. Interestingly, in the cyclin H binding site, a salt-bridge linking loop L8-9 and helix H10, mediated by residues D340 and R387, is stronger in the S371E conformational ensemble, confirming the observation made from the crystal structure. Analysis of a R387K mutant corroborated the role of the conformational equilibrium of the R387-D340 salt bridge in the phosphorylation cascade of RARγ. Simulations of the R387K mutant showed an altered conformational equilibrium of the K387-D340 salt bridge with a weakening of the interactions between these two residues, but without large disruption of the structure. The R387K mutant also depicted altered dynamics around the phosphorylation site (S371), by alteration of the angle between helices.

Finally, experimental validation indicated that modulation of the conformational equilibrium of the RARγ LBD by mutating S371 or R387 has clear effects on the phosphorylation of the N-terminal region of the receptor, as assessed by immunoprecipitation with phospho-specific antibodies. Indeed the phosphomimetic S371E mutation enhanced NTD phosphorylation in response to RA addition, while the S371N mutation (which abolishes phosphorylation) showed no response to RA. The interesting point is that the R387K mutation (which abolishes the reinforcement of the salt bridge with D340 observed in the S371E mutant) also has a clear non-response. This suggests that, even though they are separated by the long helix H9, the two residues, S371 and R387, are dynamically coupled and that the formation of the R387-D340 salt bridge is important for the phosphorylation cascade and the subsequent phospho-regulation of the NTD.

Altogether, our results show that mutations affecting the electrostatic interactions either around the C-terminal part (S371E) or around the N-terminal (R387K) of helix H9, affect the dynamics of the conformational ensemble of RARγ. Allosteric regulation by alteration of the population of conformational states has been demonstrated in several cases [60–64] and the importance of ionic pair interactions in allostery has also been shown previously in several computational studies [64, 65]. Most importantly, our results are coherent with our recent study of the RARα subtype, where we showed that phosphorylation of S369 (which corresponds to S371 in RARγ) also activates an allosteric communication pathway that does not rely on any significantly large conformational changes [66]. In this previous work, we showed that the allosteric communication observed in the RARα subtype implicated loop L8-9, which corresponds to the cyclin H docking site, allowing the binding of the cyclin H/cdk7/CAK sub-complex of the general transcription factor TFIIH and the phosphorylation by cdk7 of the serine residue located in the NTD (S77) [27, 28, 57] (Fig 1). The present study demonstrated that a similar mechanical mechanism is valid for the RARγ nuclear receptor. Thus, although the electrostatic interaction network affected by the phosphorylation is not strictly conserved between RARα and RARγ, the mechanisms observed for the two sub-types follow a similar scenario. Difference in mechanistic details, besides differences in sequence, could also be linked to the presence of a coactivator peptide in the RARα study, which is not present in this work.

To obtain a full molecular picture of the phospho-regulation of the RARs LBDs, it would be highly desirable to have an experimental structure of its complex with cyclin H and/or the full CAK complex. However, the transient nature of these complexes did not allow such a structural determination so far. Nevertheless, the coherent picture that has emerged from comparing RARα and RARγ, as well as the convergent mechanistic features identified in the structural, computational and mutation work, gives us confidence that phosphorylation of a serine residue located in loop H9-H10 of RARs propagates allosteric signals through redistribution of salt bridges networks and mechanical features around helix H9.

## Supporting Information

S1 Fig Time evolution of the Cα-RMSD along the MD simulations of RARγ wild-type and S371E and R387K mutants. Each run is represented with a different color line.
(TIF)

S2 Fig Distance distributions between nitrogen of R369 and oxygen atoms of E/S370 side chains. Distances are calculated for the WT (A) and S371E (B) simulations, vertical lines represent the calculated averages. Shown in inset are the corresponding averages and the percentage of distances less than 4Å.
(TIF)

S3 Fig Distance distributions between nitrogen and oxygen atoms of charged side chains in the phosphorylation site. Distances are calculated between O atoms of Asp and Glu side chains and N atoms of Arg side chain. The considered distances are the ones presented in Table 1 and correspond to R368-D324 (first panel), R368-E327 (second panel), R369-E322 (third panel) and R369-E327 (fourth panel). All distances were computed in the WT (A), S371E (B) and R387K (C) simulations. Vertical lines represent the calculated averages. Shown in inset are the corresponding averages and the percentage of distances less than 4Å.
(TIF)

S4 Fig Distance distributions between nitrogen and oxygen atoms of charged side chains in the cyclin H docking site. Distances are calculated between O atoms of D side chain and N atoms of R and K side chains, as discussed in Table 1. First panel corresponds to the R/K387-D340 interaction and second panel to R341-D269 interaction for the WT (A), S371E (B) and R387K (C) simulations. Vertical lines represent the calculated averages. Shown in inset are the corresponding averages and the percentage of distances less than 4Å.
(TIF)

S1 Table Data collection and refinement statistics. (PDF)
