# Supplementary figures and images for: Allosteric Regulation in the Ligand Binding Domain of Retinoic Acid Receptorγ
Source: PLoS One. 2017 Jan 26;12(1):e0171043. doi: 10.1371/journal.pone.0171043 (PMC5268703; doi:10.1371/journal.pone.0171043)

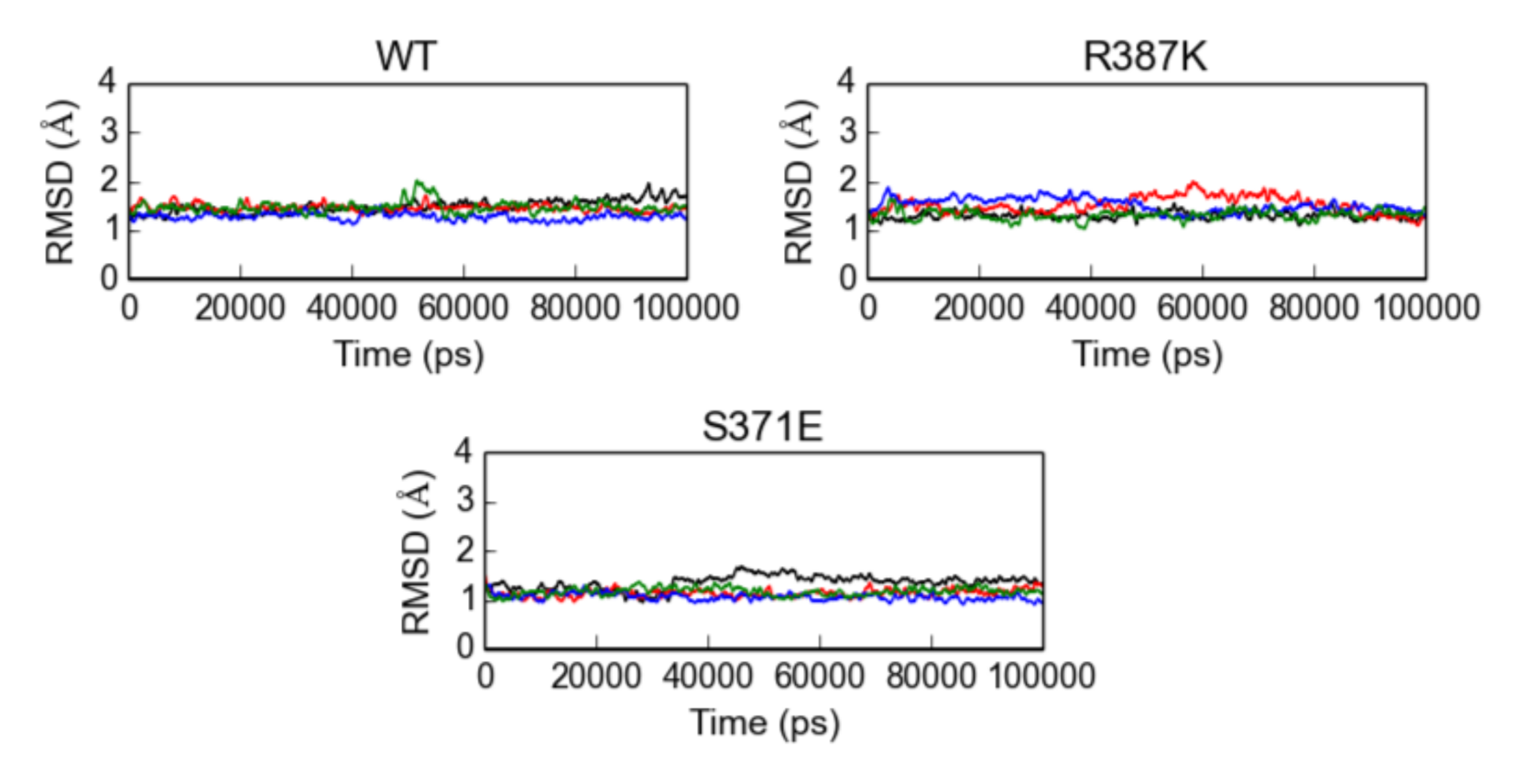

Supplement: S1 Fig — Each run is represented with a different color line. (TIF) [file pone.0171043.s001.tif]

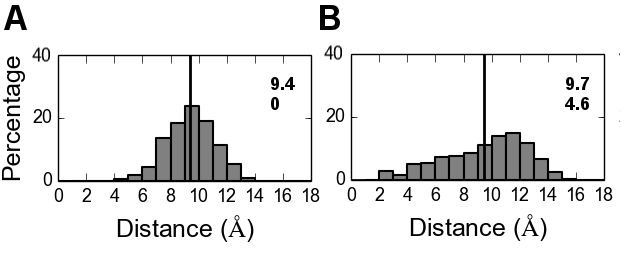

Supplement: S2 Fig — Distances are calculated for the WT (A) and S371E (B) simulations, vertical lines represent the calculated averages. Shown in inset are the corresponding averages and the percentage of distances less than 4Å. (TIF) [file pone.0171043.s002.tif]

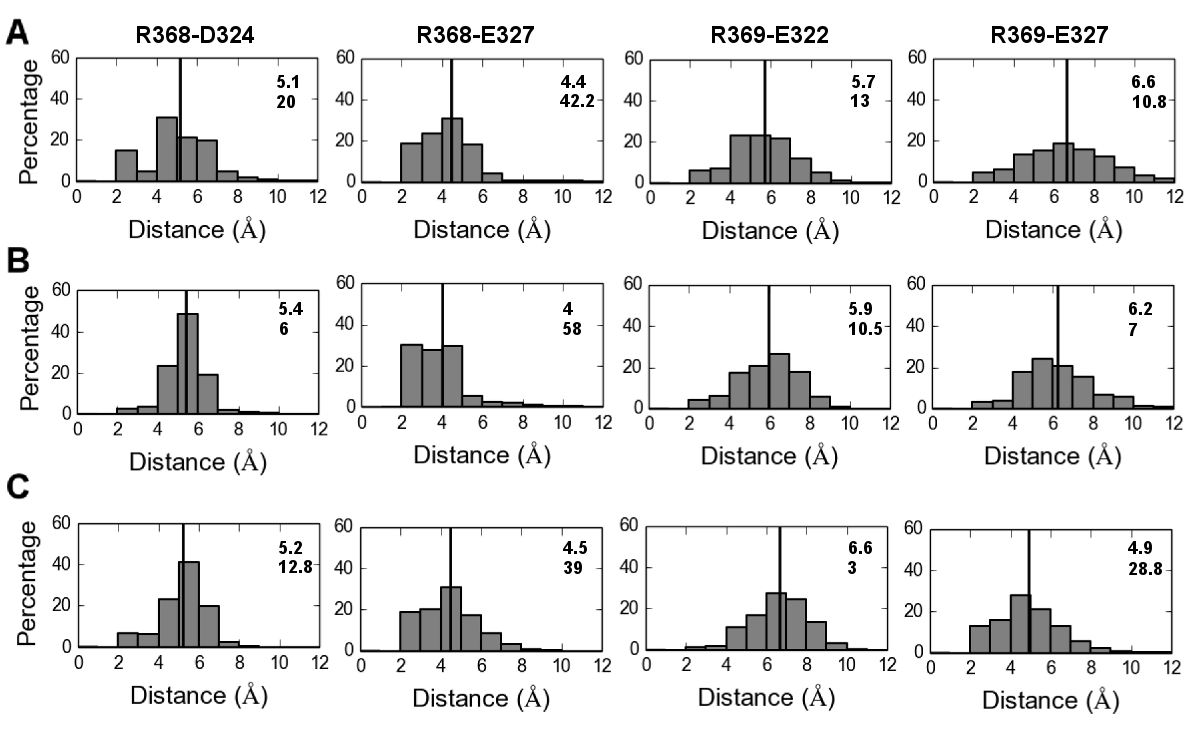

Supplement: S3 Fig — Distances are calculated between O atoms of Asp and Glu side chains and N atoms of Arg side chain. The considered distances are the ones presented in Table 1 and correspond to R368-D324 (first panel), R368-E327 (second panel), R369-E322 (third panel) and R369-E327 (fourth panel). All distances were computed in the WT (A), S371E (B) and R387K (C) simulations. Vertical lines represent the calculated averages. Shown in inset are the corresponding averages and the percentage of distances less than 4Å. (TIF) [file pone.0171043.s003.tif]

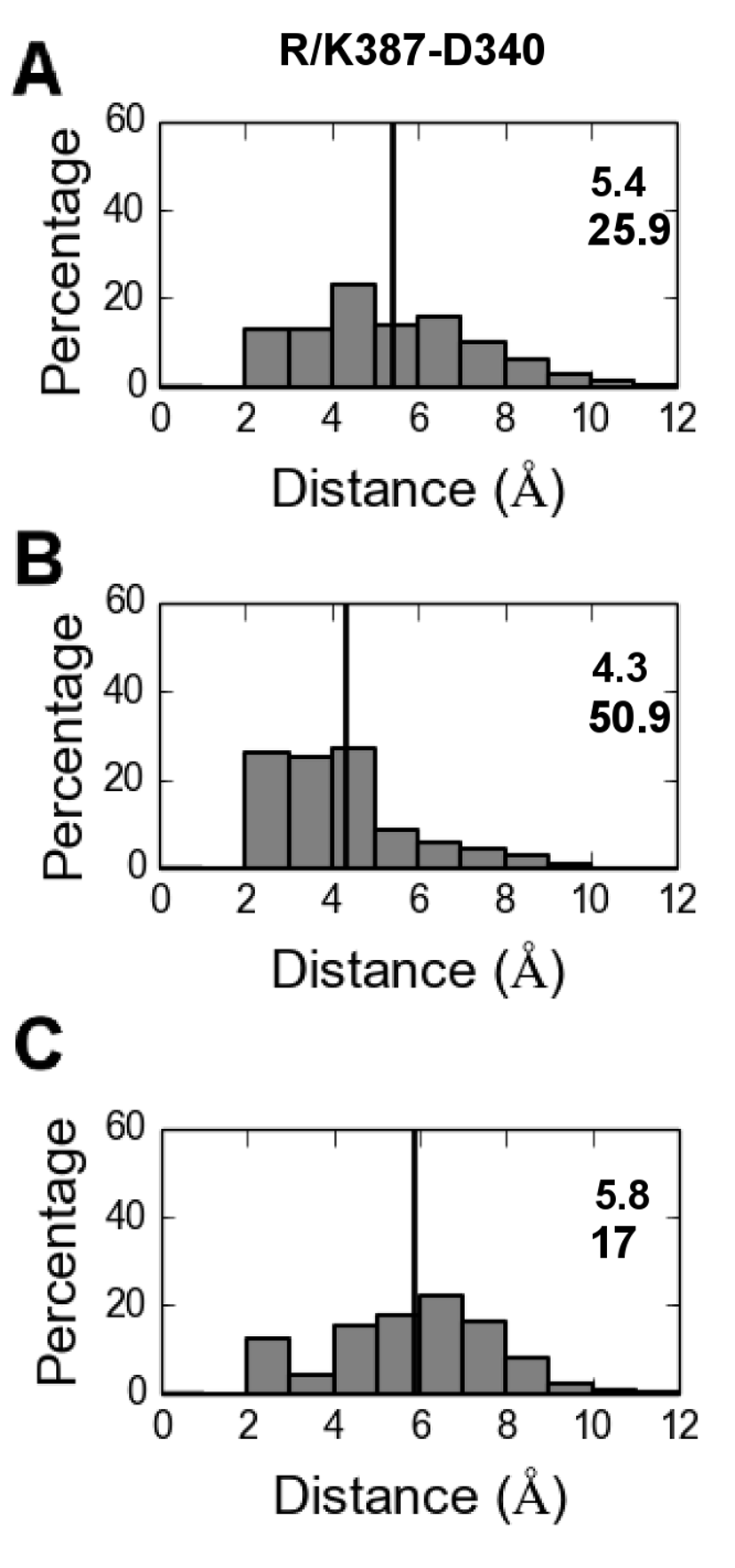

Supplement: S4 Fig — Distances are calculated between O atoms of D side chain and N atoms of R and K side chains, as discussed in Table 1. First panel corresponds to the R/K387-D340 interaction and second panel to R341-D269 interaction for the WT (A), S371E (B) and R387K (C) simulations. Vertical lines represent the calculated averages. Shown in inset are the corresponding averages and the percentage of distances less than 4Å. (TIF) [file pone.0171043.s004.tif]
