# Supplementary material for: Allosteric Regulation in the Ligand Binding Domain of Retinoic Acid Receptorγ
Source: PLoS One. 2017 Jan 26;12(1):e0171043. doi: 10.1371/journal.pone.0171043 (PMC5268703; doi:10.1371/journal.pone.0171043)
Supplement: S1 Table — (PDF) [file pone.0171043.s005.pdf]

hRARγS371E (LBD) in complex with 9-cis retinoic acid	
Data processing	
Resolution (Å)	50-1.69 (1.72-1.69)
Crystal space group	P41212
Cell parameters (Å)	b = 59.9, c = 157.4
Unique reflections	32495 (1589)
Mean redundancy	13.4 (12.3)
Rsym (%)[a]	4.4 (31.8)
Completeness (%)	100 (99)
Mean I/σ	70.17 (11.81)
Wilson B factor (Å2)	22.1
Refinement	
Resolution (Å)	50-1.69
Number of non-hydrogen atoms	
RAR-LBD	1852
Ligands	22
Water molecules	157
RMSD bond length (Å)	0.01
RMSD bond angles (˚)	1.11
Rcryst (%)[b]	19.4
Rfree (%)[c]	23.4
Ramachandran plot (%)	
Core	94.2
Allow	5.8
Generous	0

a $R\text{sym} = 100 \times \Sigma \mathbf{h}j\ |I\mathbf{h}j - \langle I\mathbf{h}\rangle| / \Sigma \mathbf{h}j\ I\mathbf{h}j$, where $I\mathbf{h}j$ is the jth measurement of the intensity of reflection **h** and $\langle I\mathbf{h}\rangle$ is its mean value.

b $R\text{cryst} = 100 \times \Sigma||F\text{o}| - |F\text{c}|| / \Sigma|F\text{o}|$, where $|F\text{o}|$ and $|F\text{c}|$ are the observed and calculated structure factor amplitudes, respectively.

c Calculated using a random set containing 5% of observations that were not included throughout refinement [Brünger A. T., (1992) The Free R Value: a Novel Statistical Quantity for Assessing the Accuracy of Crystal Structures, *Nature* **355**, 472-474].

S1 Table: Data collection and refinement statistics
